# The Mechanisms of Changes in Storage Substances and Hormone Levels During Artificial Aging of Different Varieties of Perilla

**DOI:** 10.3390/cimb48050484

**Published:** 2026-05-06

**Authors:** Yang Hang, Jinwei Pan, Xiang Yi, Jianqin Zhang, Feng Bin, Huilin Li, Weihong Lin, Tingting Yuan, Erru Yu

**Affiliations:** 1 Guizhou Academy of Agricultural Sciences, Guizhou Institute of Oil Crops, Guiyang 550009, China; 2 Guiyang Municipal Bureau of Agriculture and Rural Affairs, Guiyang Rural Revitalization Service Center, Guiyang 550081, China

**Keywords:** perilla, seed aging, antioxidant system, storage substances, hormones

## Abstract

Seed aging is a key issue that affects the preservation of germplasm resources and crop production. At present, the anti-aging properties of perilla seeds and the mechanisms of differences among varieties have not been clearly reported. This study aims to analyze the mechanisms of changes in storage substances and hormone levels in different varieties of perilla during the artificial aging process. The results show that seed aging can significantly reduce the activity of antioxidant enzymes in perilla seeds, decrease the contents of storage nutrients such as soluble proteins, soluble sugars, and oil content, reduce the accumulation of unsaturated fatty acids such as oleic acid, linoleic acid, and linolenic acid, and significantly decrease the contents of endogenous Gibberellic acid (GA_3_) and Indole-3-acetic acid (IAA). Meanwhile, the levels of hydrogen peroxide (H_2_O_2_) and Malondialdehyde (MDA) increased significantly, while the contents of saturated fatty acids such as palmitic acid and stearic acid rose, and the contents of endogenous Abscisic acid (ABA), Jasmonic acid (JA), Salicylic acid (SA), and Trans-zeatin riboside (TZR) were significantly upregulated. There are significant genotype differences in the tolerance of different perilla varieties to seed aging. The sensitivity of Shiban Perill (S23014) to aging stress is significantly higher than that of Qisu No. 2 (S23017). This study has confirmed that seed aging has adverse effects on the germination of perilla seeds by down-regulating the activity of antioxidant enzymes, reducing the accumulation of storage nutrients, and disrupting the balance of endogenous hormones. The research results provide an important theoretical basis for the preservation of perilla seed germplasm resources and the selection of anti-aging varieties.

## 1. Introduction

Perilla (*Perilla frutescens* (L.) Britt.), an annual herbaceous plant belonging to the Lamiaceae family, is a quintessential medicinal and edible plant. It is widely distributed across East Asian countries such as China, South Korea, Japan, and Vietnam [1]. Research has now confirmed that perilla possesses multiple pharmacological activities, including antibacterial and anti-inflammatory effects, antioxidant properties, anticancer activity, antithrombotic effects, lipid-lowering properties, and the ability to enhance immunity and memory [2]. Perilla seeds contain approximately 40% oil by weight. The oil is primarily composed of unsaturated fatty acids with a low proportion of saturated fats, and over 60% of its fatty acids are alpha-linolenic acid. It is hailed as land-based deep-sea fish oil [3]. Due to its rich content of bioactive components such as alpha-linolenic acid and phenolic compounds, it holds significant application value in the food, pharmaceutical, and industrial sectors [4]. Seeds serve as the core foundational material for genetic research and breeding applications. The preservation quality and storage stability of perilla germplasm resources directly impact the effectiveness of variety promotion and the progress of industrial development [5]. However, perilla seeds are typical oilseeds, and their high unsaturated fatty acid content makes them prone to natural aging during storage, leading to a decline in seed viability [6]. Artificial aging technology serves as a method for investigating seed deterioration mechanisms. It replicates the physiological and biochemical changes occurring during natural aging processes, providing a reliable research foundation for analyzing seed resistance to aging [7].

Seed aging is the irreversible decline in seed viability, quality, and physiological performance from high to low levels during storage [6]. During this process, seed viability, quality, and nutritional value gradually decline over time [8]. Research has found that even under standard gene bank storage conditions, seed germination rates decline at an annual rate of 0.2% to 0.3% [9]. Under artificial aging conditions, the seeds of crops such as maize (*Zea mays* L.) [10], soybean (*Glycine max* L.) [11], and sunflower (*Helianthus annuus* L.) [12] exhibit reduced germination rates, abnormal seedling development, weakened vigour, and diminished stress tolerance. Seed aging frequently results in membrane lipid peroxidation, degradation of soluble sugars and proteins, disruption of the antioxidant system’s equilibrium, and hormonal imbalance [13,14]. Reactive oxygen species (ROS) are signaling molecules that play a crucial role in plant growth and development, hormone signaling, and responses to biotic and abiotic stresses [15]. ROS-induced membrane lipid peroxidation compromises the integrity of cell membranes, DNA, and RNA. Concurrently, the formation of reducing sugars and their reaction with proteins alters the spatial structure of proteins and enzymes, diminishing enzymatic activity and ultimately resulting in the loss of seed viability [14]. The antioxidant system constitutes one of the protective mechanisms by which plants respond to abiotic stress. Enzymes such as superoxide dismutase (SOD), catalase (CAT), ascorbate peroxidase (APX), and glutathione reductase (GR) effectively scavenge ROS, thereby maintaining redox homeostasis [16]. In studies of rice [17], maize [7], soybean [15], oat [18], and chilli pepper [19] seeds, Peroxidase (POD), CAT, and APX activities exhibited a declining trend with increasing aging severity. This indicates that aging treatments cause damage to the antioxidant system, disrupting the dynamic equilibrium between ROS production and scavenging. Shresh et al. [20] indicated that seeds with high oil content are typically sensitive to storage conditions. ROS generated during seed storage can trigger oxidative stress, leading to oxidative damage in oilseed crops. During seed aging, dysfunction of the mitochondrial electron transport chain promotes ROS production, while downregulation of key genes in DNA repair pathways further exacerbates genomic damage [21]. However, it remains unclear how the antioxidant systems work in concert to regulate redox balance and maintain the activity of perilla seeds.

In addition to antioxidants, seeds are rich in macromolecular nutrients such as starch, protein, and fat, which are gradually broken down and utilised during the germination process [22]. During storage, seeds continuously respire and break down biomolecules such as sugars and triglycerides, leading to a decline in seed longevity and viability [12]. The oxidation of unsaturated fatty acids readily leads to increased permeability of oilseed cell membranes [12]. Research has demonstrated that perilla seeds stored for over two years may exhibit a 30% reduction in germination rate, alongside significant alterations in stored substances such as oil and protein content. This further confirms the pronounced impact of aging on the quality of perilla seeds [6]. Zhou et al. [23] found that impaired fatty acid degradation during the early stages of germination in mature soybean seeds is the primary cause of reduced seed viability following storage. Diantina et al. [24] found that changes in seed lipid composition during ageing are associated with the loss of seed viability in numerous plant species. Plant hormones also play a crucial role in regulating seed aging and viability [25].

Endogenous plant hormones are key regulators of seed longevity [26]. During seed deterioration, Abscisic acid (ABA) typically increases, promoting dormancy and inhibiting germination [27]. However, Gibberellic acid (GA_3_) levels tend to decline, reducing the ability of seeds to break dormancy and initiate growth [28]. As wheat [25], onion [29], and rice [30] seeds undergo increasing aging, their levels of GA_3_ and indole-3-acetic acid (IAA) decrease, while ABA content rises. This leads to a reduction in the GA_3_: ABA ratio, thereby inhibiting seed germination. The balance between ABA and GA_3_, and their interactions with ROS and antioxidant systems, are crucial in determining seed longevity and germination success [31].

Currently, there is limited research on the dynamic changes in storage substances and endogenous hormones in perilla seeds subjected to artificial aging. Understanding the aging mechanisms during seed storage is crucial for the preservation of perilla resources. This study utilized two perilla seed resources as materials. Through aging treatments at different time points, it investigated the patterns of storage substance changes and endogenous hormone variations in perilla seeds, elucidating the key physiological mechanisms by which aging leads to reduced seed viability.

## 2. Materials and Methods

### 2.1. Test Materials

This study selected 17 *Perilla frutescens* resources from five provinces within China (Table 1), comprising 12 samples from Guizhou Province, one from Gansu, one from Hebei, two from Shaanxi, and one from Shanxi. Seventeen test materials showed no seedling loss over the consecutive years of 2023 and 2024, exhibiting robust growth. These materials were preserved and supplied by the Oil Crops Research Institute of the Guizhou Academy of Agricultural Sciences.

### 2.2. Experimental Design for Perilla Resource Screening

This experiment was performed in 2023 at the Physiological and Biochemical Laboratory of the Guizhou Oil Crops Research Institute. Artificial aging treatment of perilla seeds was implemented in accordance with the method reported by Lin et al. [15]. Perilla seeds were preserved for one month after harvest prior to the implementation of aging trials. Each seed aging chamber was loaded with 50 g of *Perilla frutescens* seeds, and the seeds were treated in a sealed environment under high temperature (42 ± 2 °C) and high humidity (90% RH) for 0 h, 24 h, 48 h, and 72 h, respectively. Following the respective aging treatments, all seeds were allowed to dry at room temperature for two days prior to the initiation of germination tests. The germination tests were conducted in strict compliance with the International Seed Testing Schemes [32]. Briefly, seeds were subjected to disinfection using a 0.1% sodium hypochlorite solution for 5 min, after which they were rinsed thoroughly with clean water on five separate occasions. First, the seeds were disinfected with a 0.1% sodium hypochlorite solution for 5 min, and then rinsed with clean water five times. The seeds were evenly distributed in a Petri dish lined with two layers of filter paper moistened with 2 mL of distilled water. The dish was then placed in a constant-temperature incubator set at 25 °C with 85% relative humidity, under a photoperiod of 16 h light/8 h dark. Each dish contained 100 seeds and the experiment was repeated three times. Two representative perilla germplasm resources were selected on the basis of seed germination rate, and the screened germplasm resources were applied in the subsequent aging, germination, and related physiological and biochemical tests.

### 2.3. Design of Perilla Seed Aging Trials

Through initial screening of perilla resources, S23014 and S23017 were selected as experimental materials. Aging tests were conducted following the test method described in Section 2.1, and four treatment groups were set up: T0: 0 h of aging treatment; T1: 24 h of aging treatment; T2: 48 h of aging treatment; T3: 72 h of aging treatment. The germination rate, germination index, and vigor index were determined accordingly. On the fifth day of the germination test, 10 seedlings were collected from each replicate for the measurement of morphological indicators, including root length, seedling height, and seedling fresh weight.

### 2.4. Measurement Indicators

#### 2.4.1. Determination of Germination Rate, Germination Vigour and Germination Index

Germination rate (GR) was recorded when the radicle emerged through the seed coat and reached a length equal to that of the seed. Germination rate (GR) was calculated, and germination index (GI) and vitality index (VI) were determined.

GR (%) = Σ Gt/Nt × 100%

GI (%) = Σ(Gt/Dt)

VI (%) = S × Σ(Gt/Dt)

In this formula, Nt is defined as the total number of seeds, Gt is defined as the number of seeds germinated at day t, Dt is defined as the corresponding number of days to germination, and S is defined as the average root length (cm).

#### 2.4.2. Determination of Morphological Indicators

On the fifth day, measure root length (cm) and shoot length (mm) using a vernier caliper. On the seventh day, measure fresh weight (mg) using an electronic balance.

#### 2.4.3. Determination of Antioxidant Enzyme Activity

A certain amount of fresh perilla seed tissue was collected. The surface water and impurities were wiped off, and then the seeds were rapidly frozen with liquid nitrogen and stored in a −80 °C refrigerator. The seeds were placed in a pre-cooled mortar. The samples were fully ground into a fine powder with liquid nitrogen. Subsequently, the powdered sample was accurately weighed. Extraction buffer was added according to a tissue mass (g) to extraction volume (mL) ratio of 1:5–1:10. Extract the buffer solution as pre-cooled 1 mL of 50 mM phosphate-buffered saline (PBS) (pH 7.8). Briefly, approximately 0.1 g of tissue was homogenized with 1 mL of extraction buffer. The mixture was vortexed for 3–5 min or processed using a tissue homogenizer under ice-bath conditions, followed by centrifugation at 8000× *g* for 10 min at 4 °C. The resulting supernatant was collected and kept on ice for subsequent enzyme activity assays.

SOD activity was determined via the nitroblue tetrazolium (NBT) photoreduction method as previously described [33], with slight modifications. One unit (U) of SOD activity was defined as the amount of enzyme required to cause a 50% inhibition of the NBT photoreduction rate under the assay conditions. SOD activity was expressed as U·g^−1^ FW. POD activity was measured using the guaiacol colorimetric method [34] with minor modifications. The absorbance change was continuously recorded at 460 nm for 120 s. One unit (U) of POD activity was defined as a 0.01 increase in absorbance per minute. POD activity was expressed as U·g^−1^ FW. CAT activity was assayed following the protocol of Ekinci et al. [35] with minor modifications. The activity was calculated based on the decomposition rate of hydrogen peroxide (H_2_O_2_) at 240 nm. One unit (U) of CAT activity was defined as the amount of enzyme required to decompose 1 μmol of H_2_O_2_ per minute. CAT activity was expressed as U·g^−1^ FW. APX activity was determined by monitoring the oxidation rate of AsA at 290 nm [34], with appropriate minor modifications. One unit (U) of APX activity was defined as the amount of enzyme required to oxidize 1 μ mol of AsA per minute. APX activity was expressed as U·g^−1^ FW.

#### 2.4.4. Determination of MDA and H_2_O_2_ Content

Malondialdehyde (MDA) content was determined using the thiobarbituric acid (TBA) colorimetric method [36], with minor modifications. The concentration of MDA was calculated using the extinction coefficient and expressed as nmol·g^−1^ FW. Hydrogen peroxide (H_2_O_2_) content was measured according to the method developed by Velikova et al. [37], with minor modifications. The H_2_O_2_ content was quantified by measuring the absorbance change at 390 nm and expressed as μmol·g^−1^ FW.

#### 2.4.5. Determination of Soluble Sugars and Soluble Protein Content

Soluble sugar content was determined using the anthrone colorimetric method [38], with minor modifications, and expressed as mg·g^−1^ FW. Soluble protein content was measured via the Coomassie Brilliant Blue G-250 staining assay [39], and the results were expressed as mg·g^−1^ FW.

#### 2.4.6. Determination of Oil Content, Crude Protein and Fatty Acid Content

The NIRS DS 2500 (FUCHS LUBRICANTS LTD, Shanghai, China) [40] near-infrared spectroscopy analyzer was used to measure the oil content, crude protein content, as well as the fatty acid components such as linoleic acid, oleic acid, linolenic acid, palmitic acid, and stearic acid of the test materials.

#### 2.4.7. Determination of Endogenous Hormone Content

The determination of endogenous hormone content refers to the research method of Wu et al. [41]. Endogenous hormone content in seeds was determined using liquid chromatography–tandem mass spectrometry (LC-MS/MS). Instrumentation: AB Sciex QTRAP 6500+ mass spectrometer (AB Sciex, Framingham, MA, USA) coupled with an AB Sciex ExionLC-TMA liquid chromatography system (AB Sciex, Framingham, MA, USA). Reagents and Materials: A total of 24 plant hormone standards were used, including indole-3-acetic acid (IAA), 3-indolebutyric acid (IBA), indole-3-carboxylic acid, methyl indole-3-acetate, indole-3-carboxaldehyde, N6-isopentenyladenine (IP), isopentenyl adenosine (IPR), trans-zeatin-riboside (tZR), trans-zeatin (tZ), dihydrozeatin (DHZ), kinetin (KT), methylsalicylate, brassinolide (BR), methyl jasmonate (MeJA), dihydrojasmonic acid, N-jasmonic acid-isoleucine (JA-Ile), jasmonic acid (JA), salicylic acid (SA), abscisic acid (ABA), gibberellin A1 (GA_1_), gibberellin A3 (GA_3_), gibberellin A4 (GA_4_), gibberellin A7 (GA_7_), and 1-aminocyclopropanecarboxylic acid (ACC). Detailed spectrometric detection parameters are provided in the Supplementary Table S1. Meanwhile, 7 stable isotope-labeled internal standards (^2^H_5_-IAA, ^2^H_2_-GA_3_, ^2^H_6_-ABA, ^2^H_5_-ZT, ^2^H_5_-JA, ^2^H_4_-SA, ^2^H_3_-BR) were purchased from Shanghai Zhenzhun Biotechnology Co., Ltd. (Shanghai, China); methanol, acetonitrile, and formic acid (LC-MS grade) were purchased from Thermo Fisher Scientific (Waltham, MA, USA); ultra-pure water was prepared using a Mill-Q system (Millipore, Billerica, MA, USA); HLB adsorbent (60 mg/3 mL) was purchased from Waters Corporation (Milford, MA, USA). LC-MS/MS Analysis Conditions: 1. Liquid chromatography conditions: Chromatographic separation was performed on a Waters ACQUITY UPLC BEH C18 column (100 mm × 2.1 mm, 1.7 μm). The mobile phase consisted of solvent A (0.1% formic acid in ultra-pure water) and solvent B (0.1% formic acid in acetonitrile), with a gradient elution program: 0–2 min, 5% B; 2–10 min, 5–95% B; 10–12 min, 95% B; 12–12.1 min, 95–5% B; 12.1–15 min, 5% B. The flow rate was 0.3 mL·min^−1^, the column temperature was 40 °C, and the injection volume was 5 μL. 2. Mass spectrometry conditions: Electrospray ionization (ESI) source was used in positive/negative ion switching mode; the ion spray voltage was 5500 V (positive ion) and −4500 V (negative ion); the ion source temperature was 550 °C; the curtain gas pressure was 35 psi, ion source gas 1 (GS1) and gas 2 (GS2) were both 55 psi; multiple reaction monitoring (MRM) mode was adopted for quantitative analysis, with the collision energy (CE) and declustering potential (DP) optimized for each target hormone and internal standard to ensure maximum detection sensitivity. Sample Pretreatment: The seeds were ground into powder with liquid nitrogen, resuspended in ultra-pure water, and vortexed thoroughly to obtain a diluted sample. A 100 μL aliquot of the diluted sample was homogenized with 400 μL of 50% acetonitrile containing the seven stable isotope-labeled internal standards (final concentration of each internal standard was 10 ng·mL^−1^), and extracted at 4 °C for 30 min per cycle, repeating for 4 cycles. The mixture was then centrifuged at 12,000 *g* for 10 min at 4 °C. The supernatant (300 μL) was passed through the HLB sorbent (the first flow-through fraction was collected), and then the sorbent was eluted with 500 μL of 30% acetonitrile (the second flow-through fraction was collected). These two fractions were combined into the same centrifuge tube, mixed thoroughly, and then injected into the LC-MS/MS system for quantitative analysis.

### 2.5. Statistical Methods

All data were processed using Microsoft Excel 2023. Analysis of variance (ANOVA) was conducted using SPSS 20.0, with Duncan’s multiple range test employed for post hoc comparisons. Results are presented as means ± SD of the mean, with different lowercase letters denoting statistically significant differences (*p* < 0.05). Perform related analysis using Origin 2024 software. Figures in the text were generated using Origin 2024.

## 3. Results

### 3.1. Variety Selection

After subjecting 17 perilla resources to aging treatment, germination tests were conducted. Results indicate that at 0 h of aging, the top three germination rates were observed in S23017, S23014, S23005, S23108, and S23144. The germination rates of S23017 and S23014 exceeded 85%, and after 72 h of aging, they exhibited the most significant reduction compared to the non-aged samples (Table 2). Consequently, S23017 and S23014 were selected for subsequent trials.

### 3.2. The Effects of Different Aging Times on the Germination Characteristics of Different Perilla Varieties

The germination characteristics of perilla seeds are significantly regulated by aging treatment, with seed germination capacity exhibiting a marked decreasing trend as aging duration increases (Table 3). Compared to T0, the GR of S23014 were significantly reduced by 46.50%, 74.04%, and 90.99%, respectively; the GI were significantly reduced by 43.03%, 70.58%, and 87.54%, respectively; and the VI were significantly reduced by 47.88%, 96.99%, and 99.60%, respectively. Compared to T0, the GR of S23017 were significantly reduced by 31.11%, 60.37%, and 82.68%, respectively; the GI were significantly reduced by 30.92%, 60.03%, and 81.68%, respectively; and the VI were significantly reduced by 37.02%, 68.68%, and 92.77%, respectively. Significant varietal differences were observed in the reduction in GR indicators between the two perilla varieties. The decline in germination parameters was greater for S23014 than for S23017, indicating that seed aging treatment had a more pronounced effect on the germination capacity of S23014.

### 3.3. The Effects of Different Aging Times on Root Length, Bud Length and Fresh Weight of Different Perilla Varieties

By measuring the root length, shoot length, and fresh weight of seedlings, the effects of different aging periods on the morphology of perilla seedlings across varieties were analyzed (Table 4). As shown in Table 4, the growth capacity of perilla seeds diminishes with increasing aging time, manifested by reductions in root length, shoot length, and fresh weight. Compared to T0, S23014 exhibited significantly reduced root lengths of 6.74%, 34.38%, and 68.31%, respectively; significantly reduced shoot lengths of 30.21%, 63.64%, and 69.52%, respectively; and significantly reduced fresh weights of 45.38%, 67.92%, and 81.13%, respectively. Compared to T0, root length in S23017 was significantly reduced by 9.19%, 22.42%, and 62.78%, respectively; shoot length decreased by 4.86%, 16.67%, and 48.96%, respectively; and fresh weight was significantly reduced by 22.58%, 22.58%, and 61.29%, respectively. The results show that different aging times have a negative impact on the morphological indicators of the perilla seedlings. The overall decline of S23014 is greater than that of S23017. At 24 h of aging, the reduction is the smallest; at 72 h of aging, the decline is the greatest.

### 3.4. The Effects of Different Aging Times on the Antioxidant Systems of Different Perilla Varieties

**Figure 1:**
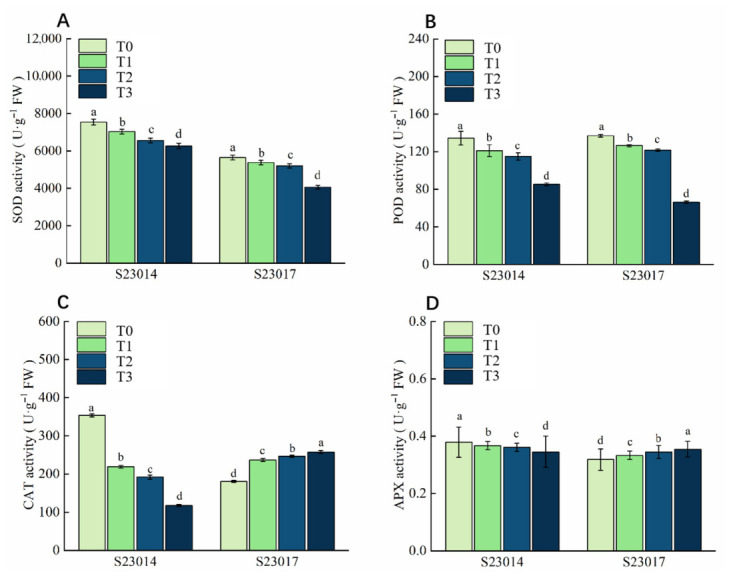
The effects of different aging times on the antioxidant systems of different perilla varieties. (**A**) SOD; (**B**) POD; (**C**) CAT; (**D**) APX. T0: 0 h of aging treatment; T1: 24 h of aging treatment; T2: 48 h of aging treatment; T3: 72 h of aging treatment. Values represent the mean ± SD of three replicate samples. According to Duncan’s multiple range test, different letters within data columns indicate significantly different values (*p* < 0.05).

After different aging times, on the seventh day of germination, the antioxidant enzyme activities in the two varieties of perilla were compared. SOD, POD, CAT, and APX activities in S23014 seeds exhibited a decreasing trend; SOD and POD activities in S23017 seeds showed a decreasing trend, while CAT and APX activities demonstrated an increasing trend (Figure 1). The SOD, POD, CAT, and APX activities of S23014 exhibited the most pronounced decline during the third stage of aging treatment. Compared to T0, the SOD activity of S23014 decreased significantly by 6.81%, 13.11%, and 16.91% respectively; compared to T0, the SOD activity of S23017 decreased significantly by 4.69%, 7.88%, and 28.08% respectively. Compared to T0, the POD activity of S23014 decreased significantly by 9.95%, 14.48%, and 36.64% respectively; compared to T0, the POD activity of S23017 decreased significantly by 7.73%, 11.12%, and 51.57% respectively. Compared with T0, the CAT activity of S23014 decreased significantly by 37.96%, 45.71%, and 66.82% respectively; Compared to T0, the CAT activity of S23017 increased significantly by 31.05%, 36.47%, and 42.69% respectively. Compared with T0, the APX activity of S23014 decreased significantly by 6.48%, 9.81%, and 18.62% respectively; compared with T0, the APX activity of S23017 increased significantly by 4.64%, 8.67%, and 11.29% respectively.

### 3.5. The Effects of Different Aging Times on Membrane Lipid Damage in Different Varieties of Perilla

**Figure 2:**
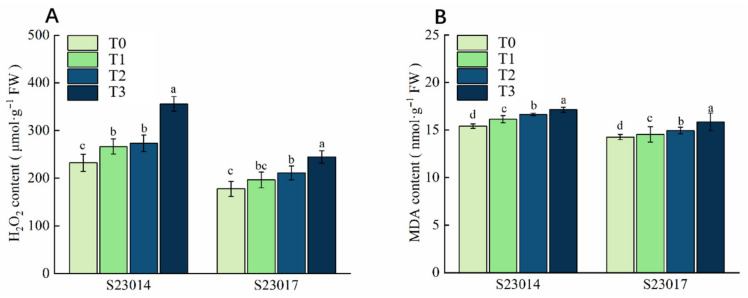
The effects of different aging times on membrane lipid damage in different varieties of perilla. (**A**) H_2_O_2_ content; (**B**) MDA content. T0: 0 h of aging treatment; T1: 24 h of aging treatment; T2: 48 h of aging treatment; T3: 72 h of aging treatment. Values represent the mean ± SD of three replicate samples. According to Duncan’s multiple range test, different letters within data columns indicate significantly different values (*p* < 0.05).

To investigate changes in ROS concentrations in artificially aged seeds, H_2_O_2_ content was measured. Following artificial aging at different time points, the H_2_O_2_ content exhibited a gradual increase in both S23014 and S23017. Compared to T0, the H_2_O_2_ content in S23014 increased significantly by 14.68%, 17.64%, and 53.29%, respectively, with higher H_2_O_2_ content observed at longer aging times. Compared to T0, the H_2_O_2_ content in S23017 increased by 10.59%, 18.88%, and 37.88%, respectively (Figure 2A). Compared with the T0, MDA content in S23014 increased significantly by 4.72%, 7.94%, and 11.24%, respectively. Compared to T0, MDA content in S23017 increased significantly by 1.97%, 4.86%, and 11.26%, respectively (Figure 2B).

### 3.6. The Effects of Different Aging Times on the Contents of Soluble Sugar and Soluble Protein in Different Varieties of Perilla

**Figure 3:**
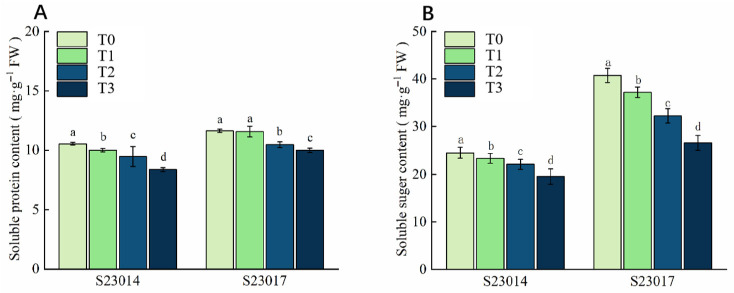
The effects of different aging times on the contents of soluble sugars and soluble proteins in different varieties of perilla. (**A**) Soluble protein content. (**B**) Soluble sugar content. T0: 0 h of aging treatment; T1: 24 h of aging treatment; T2: 48 h of aging treatment; T3: 72 h of aging treatment. Values represent the mean ± SD of three replicate samples. According to Duncan’s multiple range test, different letters within data columns indicate significantly different values (*p* < 0.05).

During the aging process, the contents of soluble sugar and soluble protein in the seeds showed a decreasing trend (Figure 3). Compared to T0, the soluble protein content of S23014 was reduced significantly by 4.19%, 10.08%, and 20.25% respectively; the soluble protein content of S23017 was reduced by 3.49%, 9.98%, and 13.90% respectively. Compared to T0, the soluble sugar content of S23014 was reduced significantly by 4.75%, 9.78%, and 20.21% respectively; the soluble sugar content of S23017 was reduced significantly by 20.90% and 34.77% respectively at 48 h and 72 h of the aging treatment. The results indicate that after the seeds aged, the degradation rates of soluble sugar and soluble protein in the seeds accelerated.

### 3.7. The Effects of Different Aging Times on the Oil Content and Crude Protein Content in Different Varieties of Perilla

**Figure 4:**
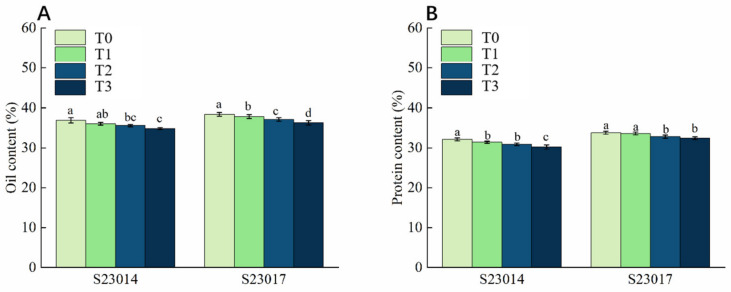
The effects of different aging times on the oil content and crude protein content in different varieties of perilla. (**A**) Oil content; (**B**) protein content. T0: 0 h of aging treatment; T1: 24 h of aging treatment; T2: 48 h of aging treatment; T3: 72 h of aging treatment. Values represent the mean ± SD of three replicate samples. According to Duncan’s multiple range test, different letters within data columns indicate significantly different values (*p* < 0.05).

To investigate the effects of aging treatment on the contents of substances in perilla seeds, this study further measured the oil content and crude protein content of the seeds. Compared to T0, the oil content of S23014 decreased by 5.7% at 72 h; compared to T0, the oil content of S23017 decreased significantly by 1.14%, 3.32%, and 5.61% respectively (Figure 4A). Compared to T0, the crude protein content of S23014 decreased by 3.9% and 6.09% at 48 h and 72 h of aging, respectively. The crude protein content of S23017 decreased by 2.99% and 4.13% at 48 h and 72 h of aging (Figure 4B). By comparing the reduction rates of the two perilla varieties, it can be seen that the crude protein reduction in S23014 at the same aging time was slightly higher than that of S23017, indicating that S23014 may be more sensitive to aging stress.

### 3.8. The Influence of Different Aging Times on the Fatty Acid Content in Different Varieties of Perilla

#### 3.8.1. Variations in Saturated Fat Content

**Figure 5:**
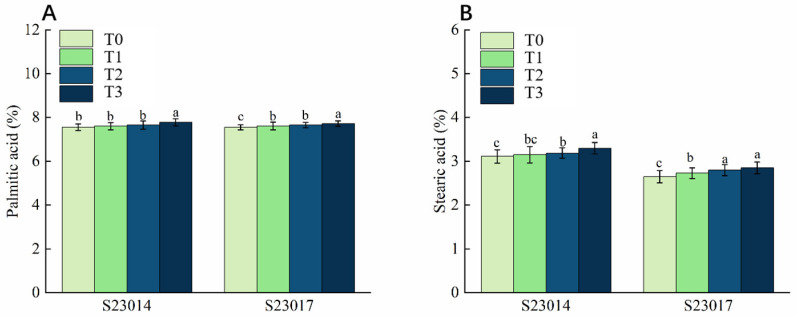
The effects of different aging times on the saturated fatty acids in different varieties of perilla. (**A**) Palmitic acid. (**B**) Stearic acid. T0: 0 h of aging treatment; T1: 24 h of aging treatment; T2: 48 h of aging treatment; T3: 72 h of aging treatment. Values represent the mean ± SD of three replicate samples. According to Duncan’s multiple range test, different letters within data columns indicate significantly different values (*p* < 0.05).

Aging treatment significantly affected the palmitic and stearic acid contents in seeds of both perilla varieties, exhibiting distinct response characteristics (Figure 5). Compared to T0, palmitic acid content in S23014 increased by 3.01% after 72 h aging, while that in S23017 increased by 2.25% after 72 h aging (Figure 5A). Unlike palmitic acid, stearic acid content exhibited a marked upward trend with increasing aging duration, with variations in the rate of increase observed between varieties. Compared to T0, stearic acid content in S23014 seeds increased by 1.18%, 2.47%, and 6.11%, respectively, at different aging times, while S23017 seeds exhibited increases of 3.14%, 5.66%, and 7.55%, respectively (Figure 5B).

#### 3.8.2. Variations in Unsaturated Fatty Acid Content

**Figure 6:**
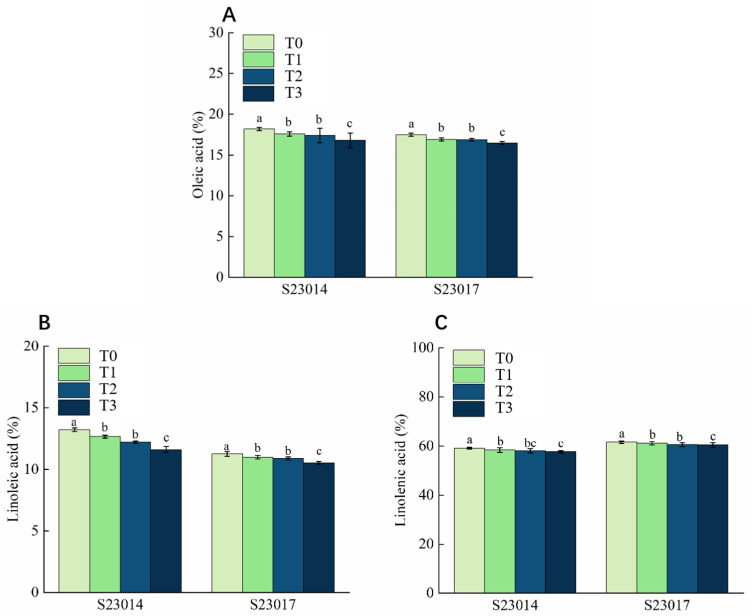
The effects of different aging times on the unsaturated fatty acids in different varieties of perilla. (**A**) Oleic acid. (**B**) Linoleic acid. (**C**) Linolenic acid. T0: 0 h of aging treatment; T1: 24 h of aging treatment; T2: 48 h of aging treatment; T3: 72 h of aging treatment. Values represent the mean ± SD of three replicate samples. According to Duncan’s multiple range test, different letters within data columns indicate significantly different values (*p* < 0.05).

To evaluate the effect of aging treatment on seed fatty acid composition, the content of unsaturated fatty acids was determined. Results indicated that the levels of all three unsaturated fatty acids (Oleic acid, Linoleic acid and Linolenic acid) exhibited a decreasing trend with prolonged aging duration. Furthermore, S23014 demonstrated significantly higher sensitivity to aging treatment than S23017 (Figure 6). Compared to T0, under different aging times, the oleic acid content in the S23014 seeds decreased by 3.37%, 4.38%, and 7.61% respectively; the oleic acid content in the S23017 seeds decreased by 2.84%, 3.55%, and 5.84% (Figure 6A). The linoleic acid content was similarly suppressed by the aging treatment. Compared to T0, the linoleic acid content in S23014 seeds decreased by 4.29%, 7.59%, and 12.08% at different aging times. In contrast, the decline in linoleic acid content in S23017 seeds was relatively moderate, showing reductions of 2.37%, 3.32%, and 6.39% compared to T0 (Figure 6B). The trend in linolenic acid content is similar to that of linoleic acid, though the overall reduction is smaller. Compared to T0, the linolenic acid content in S23014 seeds decreased by 1.25%, 1.76%, and 2.48%, respectively, under different aging times; the linolenic acid content in S23017 decreased by 0.59%, 1.01%, and 1.75%, respectively (Figure 6C).

### 3.9. The Influence of Different Aging Times on the Content of Endogenous Hormones in Different Varieties of Perilla

**Figure 7:**
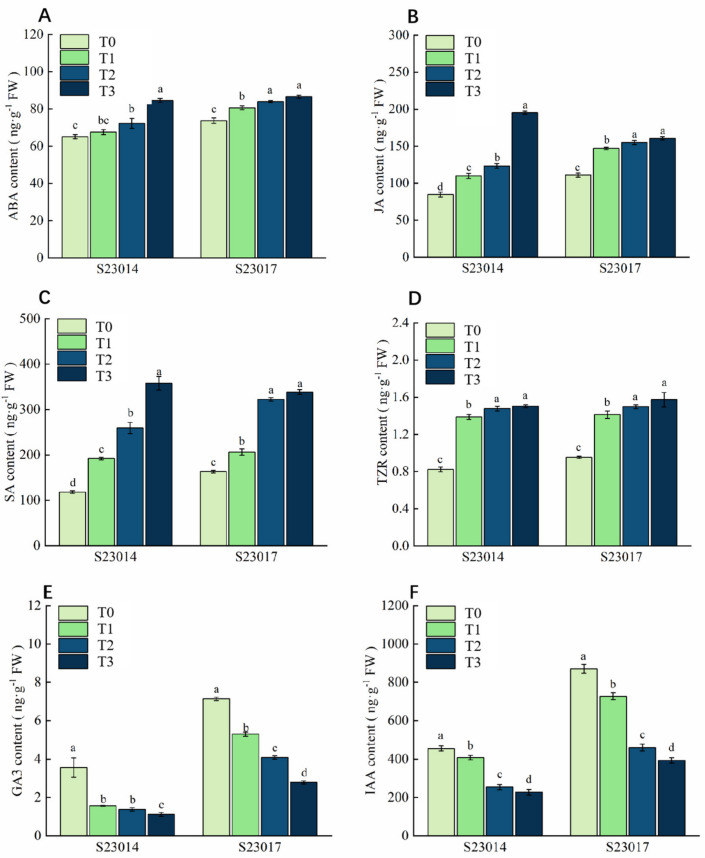
The influence of different aging times on the content of endogenous hormones in different varieties of perilla. (**A**) Abscisic acid (ABA). (**B**) Jasmonic acid (JA). (**C**) Salicylic acid (SA). (**D**) Trans-zeatin riboside (TZR). (**E**) Gibberellic acid (GA_3_). (**F**) Indole-3-acetic acid (IAA). T0: 0 h of aging treatment; T1: 24 h of aging treatment; T2: 48 h of aging treatment; T3: 72 h of aging treatment. Values represent the mean ± SD of three replicate samples. According to Duncan’s multiple range test, different letters within data columns indicate significantly different values (*p* < 0.05).

Following aging treatments at different time points, endogenous hormone levels were measured and compared between two perilla varieties. As the aging period extended, the concentrations of abscisic acid (ABA), jasmonic acid (JA), salicylic acid (SA), and trans-zeatin riboside (TZR) in both perilla varieties exhibited an upward trend, whereas those of Gibberellic acid (GA_3_) and indole-3-acetic acid (IAA) showed a gradual decline (Figure 7A–F). Compared to T0, S23014 exhibited significant increases in ABA content of 10.97% and 29.89% after 48 h and 72 h of aging, respectively; S23017 showed significant increases of 9.34%, 13.89%, and 17.44% in ABA content (Figure 7A). The JA content in S23014 exhibited a sharp increase after 72 h of aging, showing a significant increase of 130.39% compared to T0. In contrast, under the three aging treatments, S23017 exhibited significant increases in JA content of 32.46%, 39.60%, and 44.68%, respectively, compared to T0 (Figure 7B). The trend in SA content for S23014 was similar to that of JA, with SA levels rising sharply following aging treatment. Compared to T0, SA content increased significantly by 62.5%, 119.78%, and 203.01%, respectively. In S23017, no significant difference in SA content was observed between the 48 h and 72 h aging treatment groups; however, both were significantly higher than those in the T0 and the 24 h aging treatment group. Compared to T0, the SA content of S23017 increased significantly by 26.39%, 97.64%, and 107.51%, respectively, under the three aging treatments (Figure 7C). Regarding TZR content, compared to T0, S23014 exhibited significant increases of 69.28%, 80.26%, and 83.35% under the three aging treatments. Similarly, S23017 demonstrated significant TZR content increases of 48.23%, 57.07%, and 65.03% relative to T0 under the corresponding aging treatments (Figure 7D).

Compared to T0, both perilla varieties exhibited a significant downward trend in Gibberellic acid (GA_3_) content, with the reduction increasing as the degree of aging treatment intensified (Figure 7E,F). Compared to T0, GA_3_ content in S23014 was significantly reduced by 58.42%, 63.82%, and 71.03%, while S23017 exhibited significant reductions of 25.79%, 42.72%, and 60.93%, respectively. The trend in indole-3-acetic acid (IAA) content mirrored that of GA_3_, with both cultivars showing significantly lower IAA levels compared to T0. Compared to T0, the IAA content in S23014 was significantly reduced by 10.40%, 44.24%, and 50.09%; compared to T0, the IAA content in S23017 was significantly reduced by 16.43%, 47.09%, and 54.77%.

### 3.10. Correlation Analysis

**Figure 8:**
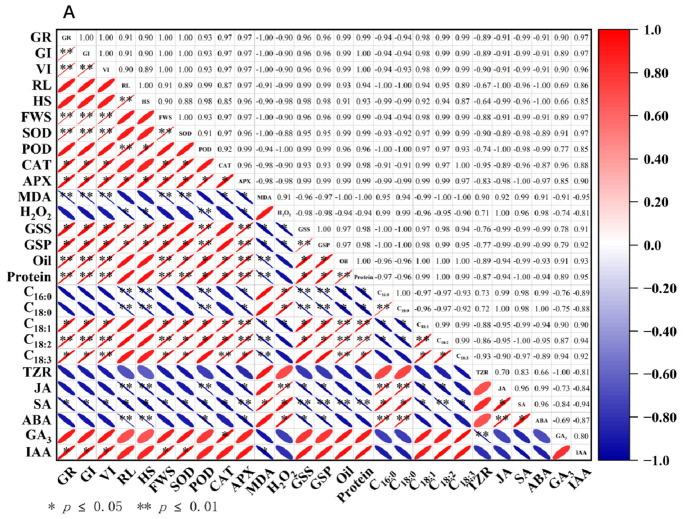

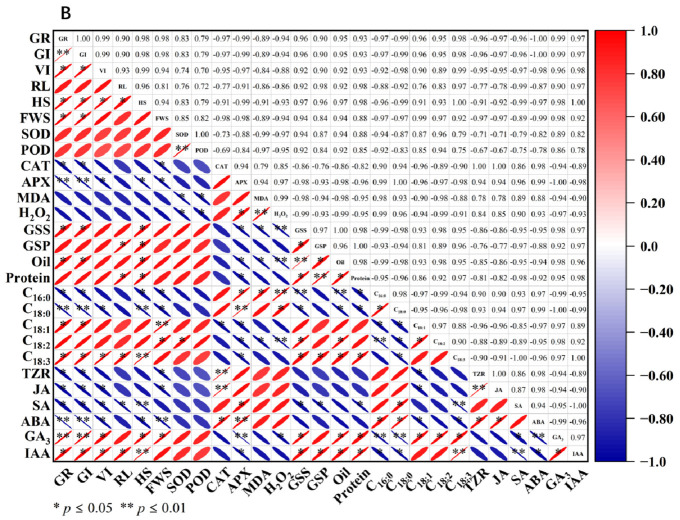
Correlation analysis between germination indices and physiological parameters of aged perilla seeds. (**A**) S23014; (**B**) S23017. GR: germination rate; GI: germination index; VI: vitality index; RL: root length; HS: seedling height; FWS: fresh weight of seedlings; SOD: Superoxide Dismutase; POD: Peroxidase; CAT: Catalase; APX: Ascorbate Peroxidase; MDA: Malondialdehyde; H_2_O_2_: Hydrogen Peroxide; GSS: soluble sugars; GSP: soluble proteins; Oil: Oil Content; Pr: Crude Protein; C_16:0_: Palmitic Acid; C_18:0_: Stearic Acid; C_18:1_: Oleic Acid; C_18:2_: Linoleic Acid; C_18:3_: Linolenic Acid; TZR: Trans-zeatin riboside; JA: Jasmonic acid; SA: Salicylic acid; ABA: Abscisic acid; GA_3_: Gibberellic acid 3; IAA: Indole-3-acetic acid.

To investigate the relationship between aging and germination indices and physiological parameters in different perilla seeds, Pearson correlation analysis was employed to examine the correlations among 27 parameters. Under different aging times, there was a significant correlation between the germination indicators of different perilla varieties and among various indicators (Figure 8). In S23014, GR exhibited extremely significant positive correlations with SOD (r = 1.00), significant positive correlations with CAT (r = 0.97) and APX (r = 0.97), and extremely significant negative correlations with MDA (r = −1.00). This indicates that oxidative stress exerts a pronounced inhibitory effect on seed germination, with SOD primarily influencing the germination of perilla seeds. GR showed extremely significant positive correlations with Oil (r = 0.99)and Pr (r = 0.99) and significant positive correlations with GSS (r = 0.96) and GSP (r = 0.96); they exhibited extremely significant positive correlations with C_18:2_ (r = 0.99) and significant positive correlations with C_18:1_ (r = 0.98). This indicates that the higher the content of storage substances and the more timely their conversion, the more readily seeds germinate successfully after aging. GR showed a significant positive correlation with IAA (r = 0.97)and a significant negative correlation with SA (r = −0.99), indicating that hormonal balance is crucial for successful seed germination following aging (Figure 8A). In S23017, GR showed positive correlations with SOD (r = 0.83) and POD (r = 0.79) and negative correlations with MDA (r = −0.89) and H_2_O_2_ (r = −0.94). They exhibited significant positive correlations with GSS (r = 0.96) and Oil (r = 0.90), indicating that germination in S23017 may depend on the effective supply of soluble sugars and lipids. GR showed a significant positive correlation with C_18:1_ (r = 0.96) and C_18:3_ (r = 0.98), a significant negative correlation with C_16:0_ (r = −0.97), and a highly significant negative correlation with C_18:0_ (r = −0.99), indicating that a higher proportion of unsaturated fatty acids is more conducive to germination. GR showed significant positive correlations with GA_3_ (r = 0.99) and IAA (r = 0.97), significant negative correlations with JA (r = −0.97) and SA (r = −0.96), and extremely significant negative correlations with ABA (r = −1.00) (Figure 8B).

## 4. Discussion

### 4.1. Effects of Artificial Ageing on Perilla Seed Germination and Seedling Morphology

Seeds form the foundation of agricultural production. The successful germination of seeds and emergence of seedlings are crucial for crop plant growth and yield formation [42]. Yin et al. [17] found that reduced seed viability was positively correlated with storage duration. Germination vigor, germination rate, germination index, and vitality index are employed to assess the degree of aging in crop seeds. In this study, following aging at different durations, the GR, GI, and VI of perilla seeds exhibited a significant decline. Furthermore, the root length, shoot length, and fresh weight of perilla seedlings were markedly inhibited, indicating that seed aging exerts an irreversible impact on seed germination and seedling emergence. This is consistent with the findings of Xu et al. [10] and Huang et al. [12]. It may be inferred that aging treatment restricts seed GR, prolongs seed emergence time, and consequently impacts the progression of seed germination.

### 4.2. Effects of Artificial Aging on the Antioxidant System of Perilla Seeds

The excessive accumulation of reactive oxygen species and lipid oxidation during seed aging is considered the primary cause of seed aging and death [43]. The role of antioxidant enzymes in maintaining optimal oxidative conditions within seeds is crucial in determining their storage capacity [7]. In this study, we investigated the relationship between the antioxidant system, ROS accumulation, and cell membrane damage with the viability of perilla seeds. SOD, POD, and CAT collectively form the enzymatic antioxidant system. Seeds are susceptible to oxidative stress during ageing; the key to ensuring seed viability lies in effectively maintaining redox homeostasis [44]. Yin et al. [17] reported that a decline in antioxidant content is responsible for the accumulation of reactive oxygen species in artificially aged seeds, thereby leading to a loss of seed viability. During seed aging, the activity of CAT and APX in rice seeds decreases [17]. Following aging of maize seeds, the activities of SOD, POD, and CAT decreased, whilst the contents of MDA and H_2_O_2_ increased. The activities of SOD and CAT exhibited a highly significant negative correlation with aging duration, whereas the contents of MDA and H_2_O_2_ showed a highly significant positive correlation with aging duration [10]. In this study, germination indices (GR, GI, and VI) exhibited a highly significant positive correlation with SOD activity, a significant positive correlation with CAT and APX activity, and a highly significant negative correlation with MDA content. Following aging treatment, the activities of SOD, POD, CAT, and APX in perilla seeds decreased, while MDA and H_2_O_2_ contents increased. This resulted in damage to the cell membrane structure of perilla seeds, leading to increased membrane permeability. These findings indicate a relationship between antioxidant enzyme activity and seed viability, with variations observed across species in the mechanisms by which antioxidant enzymes respond to aging stress [15]. These findings are consistent with those reported by Xu et al. [10] and Yin et al. [17]. Aging deteriorates the membrane structure, causing substances within the membrane to leak out. Compared to the control group, the antioxidant enzyme activity in perilla seeds decreased, weakening their capacity to eliminate toxic substances within cells.

### 4.3. Effect of Artificial Ageing on Storage Substance Content in Perilla Seeds

Oil content and fatty acid composition are key characteristics for evaluating and simulating oilseed germination. The relationship between seed fatty acid content and seed viability has been investigated in soybeans and sweet peppers. Bachleda et al. [42] reported that the content of oleic acid in soybeans was significantly and negatively correlated with the growth indicators of the seeds. Furthermore, linoleic acid content detected in sweet peppers showed a positive correlation with germination capacity; conversely, elevated palmitic acid content in seeds resulted in reduced seed viability [45]. In this study, the changes in saturated fatty acid and unsaturated fatty acid content differed during the aging process of perilla seeds. Research on maple trees [46] revealed that during seed aging, the content of linoleic acid and linolenic acid within phospholipid components significantly decreased. Phospholipid degradation and the peroxidation of unsaturated fatty acids subsequently led to membrane disruption, resulting in diminished seed germination capacity. The aging of rapeseed [47] seeds increases the content of saturated fatty acids and reduces the percentage of unsaturated fatty acids.

In this study, under aging treatment, both palmitic acid and stearic acid contents increased in the seeds of the two perilla varieties, while oleic acid, linoleic acid, and linolenic acid contents decreased with prolonged aging duration. This is consistent with the findings of Huang et al. [12]. Due to accelerated aging, the oil content and crude protein content in rapeseed have decreased [47]. This study also yielded similar results. Soluble sugars serve as the primary energy source during the early germination stage of plants [12]. Huang et al. [12] found that following seed aging, the soluble sugar content in sunflower seeds decreased, significantly reducing both seed GR and seedling emergence capacity. In this study, GSS and GSP content decreased significantly with prolonged aging duration, which may be one factor contributing to the reduced GR characteristics of perilla seeds. We hypothesize that the depletion of stored carbohydrates during aging may impede the supply of GSS to the embryo.

### 4.4. Effects of Artificial Ageing on Hormone Metabolism in Perilla Seeds

The physiological regulatory mechanisms governing seed germination are not dominated by a single plant hormone but rather involve the coordinated action of multiple hormones [25]. Plants influence physiological and biochemical processes by regulating endogenous hormone levels to adapt to adverse conditions [48]. Under stress conditions, ABA enhances plant resistance to abiotic stressors by regulating signal transduction pathways and inducing the expression of stress-responsive genes and proteins [49]. ABA and GA_3_ are two crucial plant hormones that play significant roles during seed dormancy and germination; their levels correlate closely with the degree of seed aging [49,50]. GA_3_, as a natural plant growth regulator, prevents lipid peroxidation and regulates SA [25]. Liu et al. [51] demonstrated that GA_3_ can activate endogenous hydrolase activity within seeds, thereby promoting the breakdown of stored substances. This process breaks seed dormancy and induces seed germination. In contrast, high concentrations of abscisic acid induce seed dormancy and inhibit seed germination [52,53]. Lin et al. [30] reported that the aging process significantly increased the content of ABA in rice seeds while decreasing the content of GA_3_. In this study, as aging time increased, the concentrations of ABA, JA, SA, and TZR in both perilla varieties exhibited an upward trend, whereas GA_3_ and IAA concentrations showed a gradual decline. This is consistent with findings from research conducted at Sunflower [12]. It is hypothesized that aging may influence seed viability by disrupting endogenous hormone balance.

## 5. Conclusions

Following seed aging, physiological and biochemical parameters of perilla seeds undergo alterations. The aging process disrupts the stability of the antioxidant system, diminishes the supply capacity of stored substances, and upsets the endogenous hormone balance within the seeds, thereby inhibiting seed germination and seedling growth. Following aging, antioxidant enzyme activity declines in perilla seeds, alongside reductions in soluble protein, soluble sugar, oil content, and crude protein levels. Contents of oleic acid, linoleic acid, and linolenic acid decrease, while endogenous GA_3_ and IAA concentrations diminish. Conversely, H_2_O_2_ and MDA levels increase, alongside elevated palmitic and stearic acid content, while endogenous ABA, JA, SA, and TZR concentrations rise. Furthermore, seed aging tolerance varies among perilla accessions, with S23014 exhibiting greater susceptibility than S23017. The results of this study provide a theoretical basis for revealing the aging mechanism of perilla seeds, screening anti-aging perilla varieties, and formulating storage strategies for perilla seeds.

## Figures and Tables

**Table 1:** Names and Sources of Test Materials.

Number	Material Name	Place of Origin	Material Types
S23005	Dangwu Perilla	Huaxi District, Guizhou Province	Local resources
S23014	Shiban Perilla	Huaxi District, Guizhou Province	Local resources
S23017	Qisu No. 2	Huaxi District, Guizhou Province	Variety
S23026	Huali Perilla	Kaiyang County, Guizhou Province	Local resources
S23037	Baiyi Perilla	Wudang District, Guizhou Province	Local resources
S23042	Yongjing Perilla	Xifeng County, Guizhou Province	Local resources
S23049	Jiuzhou Perilla	Xixiu District, Guizhou Province	Local resources
S23108	Jinsha Perilla	Jinsha County, Guizhou Province	Local resources
S23144	Zhijin Perilla	Zijin County, Guizhou Province	Local resources
S23296	Fengle Perilla	Sandu County, Guizhou Province	Local resources
S23308	Wengan Perilla	Wong’an County, Guizhou Province	Local resources
S23427	Meitan Perilla	Meitan County, Guizhou Province	Local resources
S23568	Taian Perilla	Tai’an County, Gansu Province	Local resources
S23571	Shiquan Perilla	Shiquan County, Shaanxi Province	Local resources
S23586	Changwu Perilla	Changwu County, Shaanxi Province	Local resources
S23593	Jizhi No. 2	Hebei Province	Variety
S23627	Xiangning Perilla	Xiangning County, Shanxi Province	Local resources

**Table 2:** The influence of different aging times on the germination rates of different perilla varieties.

Material Number	Ageing Treatment Time			
	0 h	24 h	48 h	72 h
S23005	72.00 ± 1.547 ^b^	61.67 ± 1.7638 ^b^	40.67 ± 1.7638 ^b^	30.67 ± 0.3333 ^de^
S23014	84.33 ± 0.8819 ^a^	49.67 ± 1.2019 ^cd^	25.67 ± 0.6667 ^f^	10.33 ± 1.2019 ^g^
S23017	85.33 ± 1.2019 ^a^	61.33 ± 1.2019 ^b^	35.67 ± 1.2019 ^c^	16.67 ± 0.6667 ^f^
S23026	73.67 ± 2.1858 ^b^	33.67 ± 0.8819 ^f^	34.00 ± 2.3094 ^cd^	31.00 ± 1.1547 ^de^
S23037	57.33 ± 0.8819 ^f^	25.67 ± 2.0276 ^g^	15.67 ± 0.8819 ^hi^	5.00 ± 0.5774 ^hi^
S23042	65.67 ± 0.8819 ^cd^	51.00 ± 0.5774 ^c^	47.67 ± 0.8819 ^a^	42.00 ± 1.7321 ^a^
S23049	57.00 ± 1.5275 ^f^	46.00 ± 2.3094 ^d^	44.67 ± 0.8819 ^ab^	33.00 ± 1.5275 ^cd^
S23108	72.00 ± 1.7321 ^b^	50.00 ± 2.0817 ^cd^	44.00 ± 1.0000 ^ab^	39.67 ± 1.2019 ^ab^
S23144	72.00 ± 1.1547 ^b^	70.33 ± 1.3333 ^a^	35.00 ± 1.5275 ^cd^	36.00 ± 1.1547 ^bc^
S23296	50.67 ± 2.3333 ^g^	36.67 ± 1.2019 ^ef^	31.00 ± 1.0000 ^de^	27.33 ± 1.3333 ^e^
S23380	63.00 ± 1.0000 ^de^	53.00 ± 2.5116 ^c^	20.66 ± 1.7638 ^g^	5.33 ± 1.2019 ^hi^
S23427	65.67 ± 1.2019 ^cd^	40.00 ± 1.1547 ^e^	29.67 ± 0.8819 ^e^	37.00 ± 1.5275 ^b^
S23568	69.33 ± 2.0276 ^bc^	51.33 ± 1.2019 ^c^	44.00 ± 0.5774 ^ab^	2.00 ± 1.1547 ^i^
S23571	66.67 ± 1.7638 ^cd^	50.67 ± 1.2019 ^cd^	12.33 ± 0.8819 ^i^	3.33 ± 0.8819 ^i^
S23586	60.33 ± 1.453 ^ef^	38.00 ± 1.1547 ^ef^	31.00 ± 2.0817 ^de^	28.33 ± 1.6667 ^e^
S23593	59.00 ± 2.6458 ^ef^	33.33 ± 0.8819 ^f^	7.67 ± 1.2019 ^i^	4.33 ± 1.2019 ^hi^
S23627	65.67 ± 2.3333 ^cd^	21.67 ± 1.2019 ^g^	17.33 ± 1.453 ^gh^	7.33 ± 1.2019 ^gh^

Note: Values represent the mean ± SD of three replicate samples. According to Duncan’s multiple range test, different letters within data columns indicate significantly different values (*p* < 0.05).

**Table 3:** The effects of different aging times on the germination rate, germination index and vitality index of different perilla varieties.

Material Number	Processing Number	GR (%)	GI (%)	VI (%)
S23014	T0	84.33 ± 0.8819 ^a^	17.38 ± 0.1953 ^a^	79.57 ± 2.2775 ^a^
	T1	49.67 ± 1.2019 ^b^	9.90 ± 0.2032 ^b^	41.47 ± 1.4289 ^b^
	T2	25.67 ± 0.6667 ^c^	5.11 ± 0.1223 ^c^	2.39 ± 1.0741 ^c^
	T3	10.33 ± 1.2019 ^d^	2.17 ± 0.0916 ^d^	0.32 ± 0.0178 ^d^
S23017	T0	85.33 ± 1.2019 ^a^	17.77 ± 0.1589 ^a^	82.36 ± 1.3142 ^a^
	T1	61.33 ± 1.2019 ^b^	12.28 ± 0.5655 ^b^	51.87 ± 2.2084 ^b^
	T2	35.6 ± 1.2019 ^c^	7.1 ± 0.1217 ^c^	25.8 ± 0.3163 ^c^
	T3	16.67 ± 0.6667 ^d^	3.26 ± 0.1476 ^d^	5.95 ± 0.1711 ^d^

Note: GR: germination rate; GI: germination index; VI: Vitality Index. T0: 0 h of aging treatment; T1: 24 h of aging treatment; T2: 48 h of aging treatment; T3: 72 h of aging treatment. Values represent the mean ± SD of three replicate samples. According to Duncan’s multiple range test, different letters within data columns indicate significantly different values (*p* < 0.05).

**Table 4:** The effects of different aging times on root length, bud length and fresh weight of different perilla varieties.

Material Number	Processing Number	Root Length (cm)	Sprout Length (mm)	Fresh Weight (mg)
S23014	T0	4.45 ± 0.04 ^a^	3.74 ± 0.16 ^a^	0.53 ± 0.03 ^a^
	T1	4.15 ± 0.04 ^b^	2.61 ± 0.14 ^b^	0.29 ± 0.05 ^b^
	T2	2.92 ± 0.03 ^c^	1.36 ± 0.10 ^c^	0.17 ± 0.01 ^c^
	T3	1.41 ± 0.01 ^d^	1.15 ± 0.01 ^d^	0.10 ± 0.08 ^d^
S23017	T0	4.63 ± 0.05 ^a^	2.88 ± 0.17 ^a^	0.31 ± 0.05 ^a^
	T1	4.22 ± 0.13 ^b^	2.74 ± 0.16 ^a^	0.24 ± 0.02 ^b^
	T2	3.63 ± 0.07 ^b^	2.40 ± 0.26 ^a^	0.24 ± 0.06 ^b^
	T3	1.83 ± 0.06 ^c^	1.47 ± 0.34 ^b^	0.12 ± 0.01 ^c^

Note: T0: 0 h of aging treatment; T1: 24 h of aging treatment; T2: 48 h of aging treatment; T3: 72 h of aging treatment. Values represent the mean ± SD of three replicate samples. According to Duncan’s multiple range test, different letters within data columns indicate significantly different values (*p* < 0.05).

## Data Availability

The data presented in this study are available on request from the corresponding author. The reason is that this experiment is still conducting transcriptomic, metabolic and proteomic studies.

## Supplementary Materials

The following supporting information can be downloaded at: https://www.mdpi.com/article/10.3390/cimb48050484/s1.

## Abbreviations

germination rate	GR
germination index	GI
vitality index	VI
root length	RL
seedling height	HS
fresh weight of seedlings	FWS
Superoxide Dismutase	SOD
Peroxidase	POD
Catalase	CAT
Ascorbate Peroxidase	APX
Malondialdehyde	MDA
Hydrogen Peroxide	H_2_O_2_
Soluble sugars	GSS
Soluble proteins	GSP
Oil Content	Oil
Crude Protein	Pr
Palmitic Acid	C_16:0_
Stearic Acid	C_18:0_
Oleic Acid	C_18:1_
Linoleic Acid	C_18:2_
Linolenic Acid	C_18:3_
Trans-zeatin riboside	TZR
Jasmonic acid	JA
Salicylic acid	SA
Abscisic acid	ABA
Gibberellic acid 3	GA_3_
Indole-3-acetic acid	IAA
Phosphate-buffered saline	PBS
Nitroblue tetrazolium	NBT
Unit	U
Reactive oxygen species	ROS
