# Supplementary material for: The Mechanisms of Changes in Storage Substances and Hormone Levels During Artificial Aging of Different Varieties of Perilla
Source: Curr Issues Mol Biol. 2026 May 6;48(5):484. doi: 10.3390/cimb48050484 (PMC13204896; doi:10.3390/cimb48050484)
Supplement: Supplementary file 1 [file cimb-48-00484-s001.zip › cimb-4194980-supplementary.pdf]

Table Spectrometric Detection Parameters of Plant Hormones

NO.	Component Name	Q1	Q3	RT[
1	Indole-3-acetic acid	176	130	4.56
2	3-Indolebutyric acid	204	186	5.07
3	Indole-3-carboxylic acid	160	116	4.38
4	Methyl indole-3-acetate	190	130	5.42
5	Indole-3-carboxaldehyde	146	118	4.53
6	N6-Isopentenyladenine	204	136	3.99
7	Isopentenyl adenosine	336	204	4.21
8	trans-Zeatin-riboside	352	220	3.54
9	trans-Zeatin	220	136	2.54
10	Dihydrozeatin	222	136	2.71
11	Kinetin	216	81	3.81
12	Methylsalicylate	151	91	6.07
13	Brassinolide	481.7	445.5	6.02
14	Methyl jasmonate	225	151	6.46
15	Dihydrojasmonic acid	211	59	5.46
16	N-Jasimonic acid-isoleucine	324	278	5.47
17	(±)-Jasmonic acid	209	59	5.16
18	Salicylic acid	137	93	4.71
19	Abscisic acid	263	153	4.72
20	GibberellinA1	347	273	4.12
21	GibberellinA3	345	143	4.1
22	GibberellinA4	331	313	5.39
23	GibberellinA7	329	223	5.31
24	1-Aminocyclopropanecarboxylic acid	102	56	1.09
25	Indole-3-acetic acid-D4	180	133	4.56
26	Jasmonic Acid-D5	214	62	5.16
27	N6-Isopentenyladenine-D6	210.2	137.2	3.99
28	Dihydrozeatin-D3	225	136.2	2.71
29	GibberellinA1-D4	351	307	4.12
30	Salicylic acid-D4	141	97	4.71
31	Abscisic acid-D6	269	159	4.72

Note: Q1 represents the parent ion, Q3 represents the daughter ion, RT indicates the retention time, DP denotes the de-isolation voltage, and CE stands for the collision voltage.
